# The Airborne Metagenome in an Indoor Urban Environment

**DOI:** 10.1371/journal.pone.0001862

**Published:** 2008-04-02

**Authors:** Susannah G. Tringe, Tao Zhang, Xuguo Liu, Yiting Yu, Wah Heng Lee, Jennifer Yap, Fei Yao, Sim Tiow Suan, Seah Keng Ing, Matthew Haynes, Forest Rohwer, Chia Lin Wei, Patrick Tan, James Bristow, Edward M. Rubin, Yijun Ruan

**Affiliations:** 1 Department of Energy (DOE) Joint Genome Institute, Walnut Creek, California, United States of America; 2 Genomics Division, Lawrence Berkeley National Laboratory, Berkeley, California, United States of America; 3 Genome Institute of Singapore, Singapore, Republic of Singapore; 4 Environmental Health Institute, National Environment Agency, Singapore, Republic of Singapore; 5 Department of Microbiology, National University of Singapore, Singapore, Republic of Singapore; 6 Biology Department and Center for Microbial Science, San Diego State University, North Life Sciences, San Diego, California, United States of America; Pasteur Institute, France

## Abstract

The indoor atmosphere is an ecological unit that impacts on public health. To investigate the composition of organisms in this space, we applied culture-independent approaches to microbes harvested from the air of two densely populated urban buildings, from which we analyzed 80 megabases genomic DNA sequence and 6000 16S rDNA clones. The air microbiota is primarily bacteria, including potential opportunistic pathogens commonly isolated from human-inhabited environments such as hospitals, but none of the data contain matches to virulent pathogens or bioterror agents. Comparison of air samples with each other and nearby environments suggested that the indoor air microbes are not random transients from surrounding outdoor environments, but rather originate from indoor niches. Sequence annotation by gene function revealed specific adaptive capabilities enriched in the air environment, including genes potentially involved in resistance to desiccation and oxidative damage. This baseline index of air microbiota will be valuable for improving designs of surveillance for natural or man-made release of virulent pathogens.

## Introduction

Modern humans spend 90% of their lives indoors [1], and the air in these enclosed spaces contains a variety of microorganisms including bacteria, fungi, and viruses, some potentially harmful to human health. While air provides an extremely harsh environment for microbial survival, airborne transmission is the predominant route for disseminating microorganisms, and malicious dispersal of infectious agents represents a potential public health risk. The origins and composition of indoor air microbiota, however, are poorly understood. Determining the overall biological diversity in the indoor atmosphere, and assessing its dynamics, is essential to facilitating the rational development of public health policies.

Airborne microbes are often attached to dust particles or water droplets from sneezes and coughs or breezes over land or bodies of water. When the water in aerosols evaporates, the microbes become droplet nuclei and clumps, most of which can stay airborne indefinitely and drift with air flows [2], [3]. While studying airborne microbes is made challenging by their low concentrations, such particles enriched with microorganisms can be collected by sampling large volumes of air through air handling units (AHU) in modern building ventilation systems, without deployment of specialized sampling devices.

Studies of cultivable airborne microbes by a variety of sampling methods have revealed numerous bacteria and fungi to be present in air [4]–[6] but likely underestimate the diversity of the air microbial community as most environmental microbes are resistant to culture. One study of outdoor air found that only 0.08% of the microscopically evident motile cells was readily cultured [7]. Culture-independent techniques such as 16S ribosomal RNA gene coding DNA (16S rDNA) analysis and metagenomic sequencing provide a less biased perspective on environmental microbes because DNA is sampled directly from the environment. In this study, we have used an AHU filtration strategy for air sample collection and performed both 16S rDNA and metagenomic analyses to characterize the airborne biological diversity in an indoor urban environment.

## Results

**Figure 1:**
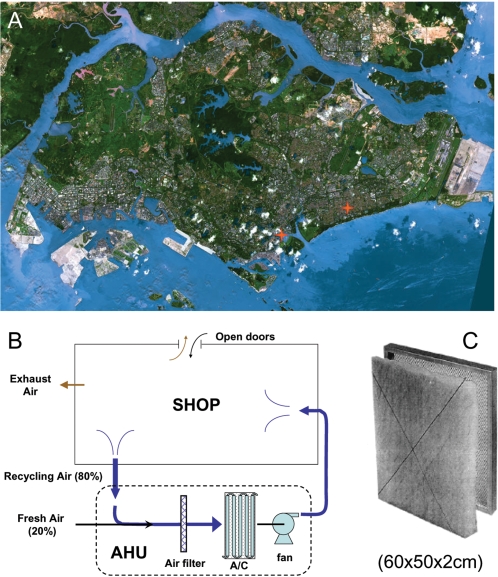
Air sampling location and device. A. The Singapore map and the two sampling locations (indicated by red stars) in Singapore. B. A schematic view of the air handling unit (AHU) filtration system used in the two buildings. C. An air panel filter (60×50×2 cm^3^) used in this study.

We collected indoor air from two shopping centers in Singapore (Figure 1A), between March and April of 2005. Using an AHU (Figure 1B), approximately 6 million cubic meters of air (80% recycled and 20% fresh) were sampled from each of the two locations for analysis. A variety of observations support the premise that the time-averaged biological populations found on the filters constitute a true sampling of the air microbiota. This includes ^3^H-thymidine and ^3^H-leucine incorporation assays revealing the majority of the cells arrested in the filters not to be actively growing (Supplement Tables S1 and S2 in Supporting Information, SI), and physical and microscopic inspections revealing no indication of active bacterial growth (data not shown). Lastly, a culture-based survey of the filter contents revealed no abnormally high growth rates for particular microorganisms, and many of the isolates obtained were gram-positive species not found to be abundant in the DNA sequence data (Tables S3 and S4 in SI).

### Indoor air microbial diversity assessed by 16S rDNA analysis

**Figure 2:**
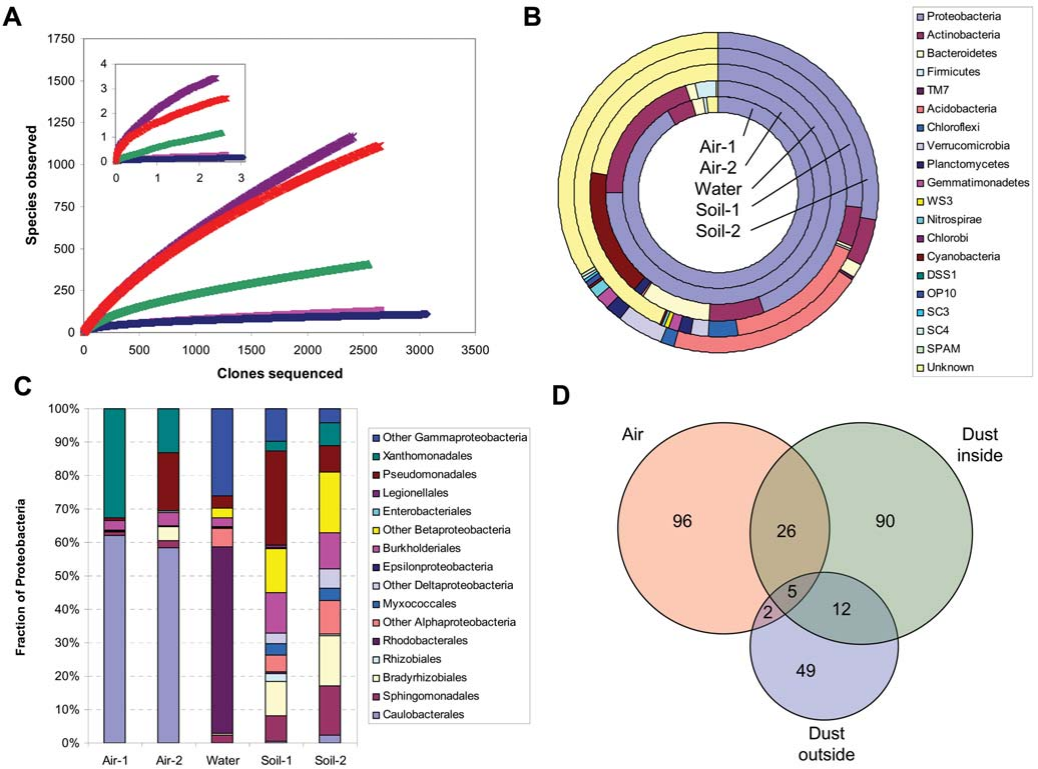
Microbial diversity in air and other local environmental samples. A. Rarefaction curves of observed phylotype diversity for Air-1 (pink line), Air-2 (dark blue), water (green), Soil-1 (purple), and Soil-2 (red). The similarity threshold is 97%. Inset: the estimated diversity in the 5 samples using the Chao1 richness estimator. B. Distribution of microbial divisions in each of the environmental samples; circles represent, from the inside out, Air-1, Air-2, Water, Soil-1, and Soil-2. C. Bacterial orders within the Proteobacteria for each sample. D. A Venn diagram of all phylotypes observed in the air and dust samples from location 2.

Phylogenetic diversity of the airborne bacteria in each location was first assessed with 16S rDNA clone sequencing (see Materials and Methods). Among the 2,659 and 3,063 high-quality 16S rDNA sequences analyzed from the two air samples of locations 1 and 2 (Air-1 and Air-2), respectively, we observed 129 and 108 distinct ribotypes, most of which (78% for Air-1 and 91% for Air-2) had close (>95% identity over >1000 bp) relatives in the Greengenes database (http://greengenes.lbl.gov/)[8] (Table 1). Based on rarefaction curves constructed from these data, the samples are estimated to contain 170 to 300 distinct species in total (Table 1, Figure 2A). This stands in contrast to multiple surveys of outdoor environments where thousands – even millions - of different species are often estimated to be present, and a significant fraction of the 16S rDNA clone sequences are novel [9]–[12].

**Table 1:** 16S rDNA analysis.

Samples	No. of 16S Sequences*	Observed			Estimated Phylotypes
		Phylotypes	Known	Novel	
Air-1	2659	129	101 (78%)	28 (22%)	286
Air-2	3063	108	98 (91%)	10 (9%)	176
Soil-1	2408	1166	484 (42%)	682 (58%)	3437
Soil-2	2648	1113	545 (49%)	568 (51%)	2592
Water	2546	407	219 (54%)	188 (46%)	1198

* all sequences are >1000 nucleotides

Estimated and observed phylotypes in different local environments at 97% identity

It is commonly presumed that airborne microbes are a random assortment of aerosolized cells from nearby primary environments such as soil and water bodies, as the air environment is inadequate to sustain growth. To compare the biological contents of air to other local habitats, we collected two top soil samples from locations close to the air-sampled buildings (Soil-1 and Soil-2) and a water sample from the Singapore River that flows next to the shopping mall in location 1, and performed 16S rDNA clone sequencing on all the samples (Table 1).

Comparisons of these data to those from air showed the microbial diversity in air to be substantially lower than that in the aquatic and terrestrial environments, which were each estimated to contain thousands of phylotypes (Table 1 and Figure 2A). The phylogenetic spectrum of organisms in the air is also very different from that of water and soil (Figure 2B and 2C; more details in Table S4 in SI), suggesting that the organisms that are abundant in air are not those that dominate nearby terrestrial or aquatic environments. Significantly, the two air samples contained more phylotypes in common with each other than with the other environmental samples (Table S4).

The most abundant airborne microbes included several species of *Brevundimonas* (56% and 44% of sequences in Air-1 and Air-2; Table S4 in SI). This cosmopolitan genus within the *Caulobacterales* has been observed almost ubiquitously in fresh and salt water, soil, sludge and oil. *Brevundimonas* species have also been cultivated from nominally sterile environments such as the space station Mir, and in clinical settings where they have been implicated in opportunistic infections [13]–[16]. Interestingly, despite the prevalence of organisms from the typically aquatic *Caulobacterales* in both air samples, only one sequence affiliated with this family was observed among the 2546 16S rDNA sequences of from the Singapore river water sample (Table S4 in SI).

16S rDNA sequence clusters closely related (many of them are ≥99% identity) to *Stenotrophomonas maltophilia* were also abundant in both the locations (29% of clones in Air-1 and 10% of clones in Air-2) (Table S4 in SI). This species, which also showed up several times in the culture-based survey (Table S3 in SI), is an environmentally widespread opportunistic pathogen frequently implicated in hospital-acquired infections [17]. There were also phylotype groups, such as *Brachybacterium*, *Acinetobacter*, and members of *Microbacteriaceae* and *Micrococcaceae*, which were frequently observed in one air sample, but not in the other (Table S4 in SI). This differential representation of phylotypes suggests that each of the two shopping centers, though harboring a shared set of abundant microbes, has its own unique indoor microbiota.

It is anticipated that air suspension particles would eventually settle to the floor. Therefore the floor sediment could reflect a fraction of the airborne organisms collected over time. We surveyed the microbial content of floor dust inside one of the buildings (Air-2) and compared it to dust immediately outside of the building using the same 16S rDNA analysis. Among the 566 and 624 clones of 16S rDNA sequences analyzed from the two samples, 5 and 7 clones from the indoor sample hit the dominant indoor air genera *Brevundimonas* and *Stenotrophomonas*, respectively, while none from the outside fell into these groups. Even more strikingly, 127 clones from the indoor dust and just 3 from the outdoor dust contained close matches to *Acinetobacter*, a genus found frequently (304 of 3063 clones) in the Air-2 clone library from this location but not that from the other location (Table S4 in SI). Overall, some 26 of the 129 phylotypes observed in Air-2 were also among the 133 phylotypes in the indoor dust, but just 7 were seen in the outside dust (Figure 2D). These data further support the hypothesis that the indoor air is a distinct habitat, and that these dominant organisms were truly present in the air and not merely on the air filters.

To identify potential origins for these airborne microorganisms, we inspected the two buildings and did not find obvious indoor habitats, such as planters or water fountains, which might serve as favorable reservoirs for these organisms. As some of the phylotype groups observed in both air samples included organisms previously observed in clinical specimens or human-inhabited environments, and included a number of human commensals and potential opportunistic pathogens (Table S7 in SI) [13], [18]–[20], it is reasonable to speculate that the human occupants of the shopping centers could contribute the airborne microbes through sneezing and coughing. We sampled a nasal swab and a saliva specimen from human subjects who had visited the shopping centers, and sequenced more than 1000 16S rDNA clones from each sample. Both of the samples were dominated by known human commensals and pathogens, such as *Corynebacteria* in the nasal swab and *Streptococcus* in saliva, but the nasal swab also contained 2 hits to the dominant air organism *Brevundimonas* (Table S4 in SI). Though more human respiratory samples should be surveyed, this overlap of observed microbes in air samples and human nasal samples suggests an active interaction between the air microbiota and human occupants in the indoor space.

In combination, the 16S rDNA analysis of the air filter samples revealed that in comparison to local microbial communities in primary habitats such as soil and water, the microbial population in air from these two buildings is of limited diversity and unique composition, indicating that the air environment harbors a community that is not a random mix of visitors from other habitats. This is consistent with a previous study indicating that certain organisms may become aerosolized preferentially [21].

### Air metagenomic analysis

We hypothesized that some organisms, such as *Brevundimonas* spp., possess features that make them amenable to air dispersal. To gain insight into the molecular mechanisms allowing these specific species but not others to be enriched in the air, we then undertook genomic shotgun sequencing to further analyze the aerogenome.

DNA isolated from the filter-trapped microbes was used to build small insert libraries for shotgun sequencing (Materials and Methods). Roughly 80 million bases total of DNA sequence were generated from the two air samples (Table S5 in SI). If a single bacterial species with a genome size of 4 Mb makes up 50% of each sample, this collection of sequences would provide a 5-fold genome coverage of the dominant bacterium in each sample, and therefore supply an informative glimpse of the previously unexplored air metagenome.

A total of 66,702 and 74,018 DNA sequence reads with more than 100 contiguous high-quality bases were generated from samples Air-1 and Air-2, respectively. 52,303 (78%) and 58,587 (79%) of these had BLASTX hits in GenBank with an e-value less than 10^−8^. By comparison, less than half of the shotgun sequencing reads from soil in a recent study had hits at this threshold [12], indicating that most air microbes are likely to be closely related to fully or partially sequenced organisms. Considering the bias towards pathogenic species in current microbial DNA sequence databases, this may suggest that many indoor air microbes originate from human-associated habitats. 88% and 72% of the hits in samples Air-1 and Air-2 respectively are to bacteria, particularly Proteobacteria, while 0.26% and 2.0% of the hits are to eukaryotes; the remainder are to archaea and sequences without an associated taxonomy (Table S6 in SI). By far the most frequent hits (28% for Air-1 and 35% for Air-2) were to the Alphaproteobacterium *Caulobacter crescentus*, a member of the same order, the *Caulobacterales*, that encompasses the *Brevundimonas* species observed repeatedly in the 16S rDNA clone library. *C. crescentus* was the only member of this order whose complete genome sequence was available in GenBank at the time of analysis. The next most abundant hits were to members of the order *Xanthomonadales* (22% for Air-1 and 8% for Air-2), which includes *Stenotrophomonas*; among the remaining hits, no more than a few percent were to any single genus. Though BLASTX hits are a poor means of species assignment [22], the prevalence of hits to these families indicates that they make up a substantial portion of the organisms trapped from air on the AHU filters. While only 153 and 132 partial 16S ribosomal DNA sequences were found within the metagenomic data from Air-1 and Air-2, they confirmed an abundance of *Caulobacterales* and *Xanthomonadales* among the studied microbes (Table S6 in SI).

Given the relatively low diversity in air, we expected some of the sequences to assemble into larger contigs. In each sample, roughly 60% of the reads assembled into contigs with reads from independent clones (Table S5 in SI). While no individual genome was covered at sufficient depth in the air filter data to achieve genome assembly, 930 and 770 contigs longer than 3 kb emerged from the Air-1 and Air-2 library assemblies, respectively, and the average length of all contigs was ∼1.5 kb (Figure S1 in SI). In both Air-1 and Air-2, the longer and deeper contigs appeared to be affiliated with the *Caulobacterales* and *Xanthomonadales* (Figure S2A in SI). Based on the 16S rDNA amplicon clone analysis, rDNA sequences present in these contigs, and the results of BLASTN and BLASTX, we believe that these were derived primarily from the *Brevundimonas* and *Stenotrophomonas* spp. documented in the 16S clone libraries. Neither of these genera had a fully sequenced representative in the public database at the time of analysis.

To further validate this observation, we conducted semi-quantitative PCR to assess the relative abundance of some of the larger contig sequences in air and other environmental samples (Figure S3 in SI). The PCR results indeed suggested that these contig sequences were only abundant in the indoor air, but not in nearby terrestrial and water samples. Importantly, when signal was detected in the soil samples, it was of lesser magnitude than in air and present only in the soil sample matched to the location of the original sequence (see Air-1 contigs 15516 and 15369, and Air-2 contig 16031).

The pathogen burden carried by indoor air has not been extensively studied outside of hospitals, and it is unclear whether organisms related to potential bioterror agents are present in our atmosphere. Reassuringly, neither the 16S rDNA clone data nor the metagenomic data contained close sequence matches to virulent pathogens such as *Bacillus anthracis, Yersinia pestis* or other species on the CDC bioterror watch list (http://www.bt.cdc.gov/agent/agentlist.asp). Some 1.5% of the assembled sequences aligned at 80–90% nucleotide identity to virulent and opportunistic pathogens, including pathogenic species of *Brucella*, *Bordetella*, *Burkholderia* and *Mycobacterium* (Table S7 in SI), but this level of sequence identity is inadequate to predict pathogenicity.

### DNA sequence functional analysis

Relatively little is known about how microbes survive the stress of becoming airborne, and whether there are genetic contributors to aerosolization or airborne dissemination. We therefore examined the metagenomic data from the air samples to identify genes and functions that are overrepresented among the air microbiota, using the orthologous groups defined by the COG (http://www.ncbi.nlm.nih.gov/COG/) [23] and STRING (http://string.embl.de/) [24] databases. We first predicted the open reading frames (ORFs) from the air DNA sequences, and then annotated the functions and assessed the frequency of the ORFs using our previously described Environmental Gene Tag (EGT) analysis [12].

**Figure 3:**
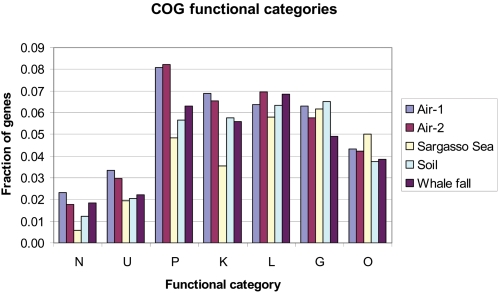
COG analysis. Predicted ORFs from the two air samples in this study and the other 3 environmental samples from previous studies were mapped to orthologous groups and broad functional categories according to the COG and STRING databases. Only functions that are more common in air than in other environments are depicted: N, Cell motility and secretion; U, Intracellular trafficking and vesicular transport; P, Inorganic ion transport and metabolism; K, Transcription; L, DNA replication, recombination and repair; G, Carbohydrate transport and metabolism; and O, Posttranslational modification.

Of the 34,984 and 44,021 predicted ORFs in the partially assembled Air-1 and Air-2 sequences, 24,733 (70.7%) and 26,897 (61.1%) of them have significant hits by BLASTP in the STRING database. On a broad functional level, genes participating in cell motility and secretion were more common in the air data than in previously characterized soil, ocean and whale fall community sequences (Figure 3). Specific gene families within these categories that were overrepresented in air filter DNA included cell membrane proteins participating in protein secretion, motility and conjugal transfer, particularly a large set of functionally related genes homologous to members of the VirB, VirD and Trb systems (Table 2). Fimbrial adhesins, whose homologs have been implicated in pathogenicity, motility and cell aggregation, also stood out as common “air community” genes; interestingly, the aggregation promoted by fimbriae in *Xanthomonas campestris* has been found to contribute to resistance to UV light and desiccation [25]. We hypothesize that these cell surface protein genes are overrepresented in the air microbial community because they improve survival in the atmosphere, but they may also facilitate colonization of human building occupants or promote adhesion of cells to the fibers of air filters.

**Table 2:** Functionally associated gene clusters overrepresented in air and their relative abundance in other environments.

COG ID	Ocean	Soil	Whale fall	Air-1	Air-2	Description
**Cytochrome bd biosynthesis**						
COG1271	0.01	0.08	0.16	0.4	0.36	Cytochrome bd-type quinol oxidase, subunit 1
COG1294	0.01	0.07	0.14	0.42	0.33	Cytochrome bd-type quinol oxidase, subunit 2
COG4988	0.05	0.01	0.13	0.39	0.41	ABC-type transport system involved in cytochrome bd biosynthesis, ATPase and permease components
COG4987	0.09	0.07	0.17	0.37	0.29	ABC-type transport system involved in cytochrome bd biosynthesis, fused ATPase and permease components
**Pilus assembly**						
COG3539	0.005	0	0	0.78	0.21	P pilus assembly protein, pilin FimA
COG3188	0	0.02	0.04	0.52	0.42	P pilus assembly protein, porin PapC
COG3121	0	0.02	0.04	0.31	0.64	P pilus assembly protein, chaperone PapD
COG5430	0.003	0.08	0.03	0.7	0.18	Uncharacterized secreted protein
COG0582	0.08	0.11	0.21	0.24	0.37	Integrase
**Enterochelin transport**						
COG4605	0	0	0.36	0.33	0.31	ABC-type enterochelin transport system, permease component
COG4607	0	0	0.14	0.79	0.16	ABC-type enterochelin transport system, periplasmic component
COG4604	0.04	0	0.26	0.17	0.53	ABC-type enterochelin transport system, ATPase component
**Type IV secretion**						
NOG06545	0.003	0	0.04	0.24	0.72	Type IV secretory pathway, VirB1
NOG08524	0	0	0	1	0	Type IV secretory pathway, VirB2
COG3838	0	0.01	0.12	0.23	0.64	Type IV secretory pathway, VirB2
COG3702	0	0	0	0.29	0.71	Type IV secretory pathway, VirB3
COG3451	0.004	0.06	0.16	0.3	0.48	Type IV secretory pathway, VirB4
NOG08232	0.01	0	0.14	0.6	0.26	Type IV secretory pathway, VirB5
COG3704	0.01	0.07	0.29	0.24	0.39	Type IV secretory pathway, VirB6
COG3736	0.01	0	0.29	0.31	0.38	Type IV secretory pathway, VirB8
COG3504	0.004	0.01	0.09	0.48	0.41	Type IV secretory pathway, VirB9/TrbG
COG2948	0.01	0.03	0.17	0.26	0.52	Type IV secretory pathway, VirB10/TrbL
COG0630	0.07	0.07	0.21	0.07	0.58	Type IV secretory pathway, VirB11, and related ATPases
COG3843	0	0.01	0.13	0.23	0.63	Type IV secretory pathway, VirD2
COG3505	0.002	0.03	0.18	0.35	0.44	Type IV secretory pathway, VirD4
COG3846	0.01	0.07	0.17	0.3	0.45	Type IV secretory pathway, TrbL
COG3701	0	0.04	0.08	0.45	0.43	Type IV secretory pathway, TrbF
COG3942	0.06	0.06	0.08	0.33	0.47	Surface antigen
COG5314	0.005	0.06	0.22	0.28	0.44	Conjugal transfer/entry exclusion protein TrbJ

Also among the genes most overrepresented in the air sequences from both samples were a number of orthologous groups containing proteins involved in redox metabolism and inorganic ion metabolism (Figure 3, Table 2). These include a set of genes necessary for the biosynthesis of cytochrome bd, a terminal oxidase known to be important for survival of oxidative stress and iron deprivation [26] as well as virulence in some pathogens [27], [28]. Genes involved in iron transport and metabolism, particularly siderophore synthesis, sensing and uptake, were also prevalent in air microbes (Table 2). Not surprisingly, many poorly characterized and uncharacterized genes were found exclusively or predominantly in air data relative to sequence from previously examined outdoor environments.

## Discussion

This metagenomic analysis of microbial species in a densely populated urban indoor atmosphere demonstrates that the organisms in air are distinct from those found in surrounding outdoor environments and possess unique genomic features. The considerable similarity observed in the indoor air organisms and metagenomes derived from two closely separated localities suggests that indoor air microbial communities share organisms and genetic features in common, although the primary habitats for these organisms are not entirely clear but likely include humans themselves.

Based on our functional assessments, it appears that the major stresses encountered by air microbiota may be iron limitation, oxidative damage and desiccation, all of which could reasonably be expected in indoor air. Our results also imply that the indoor air environment exerts a specific selective influence on microbes, so as to enrich certain organisms in the air. Sequences suggestive of opportunistic pathogens as well as virulence-associated genes are also common in the air DNA, indicating that infectious agents, but not virulent pathogens, are likely present in everyday air. These findings suggest that the resident microbes in the indoor atmosphere have been selected for an indoor life cycle, part of which is spent in the air.

This baseline characterization of air microbiota provides an in-depth glimpse of an everyday environment closely encountered by the general public. However, the airborne microbiota may be dynamic and sensitive to changes by direct and indirect factors ranging from outside climate variation to indoor occupants and micro-niche establishment [29], and further analysis of sub-compartments of indoor air and different time points over a long period will be necessary to present a comprehensive picture of airborne microbiota. Our results indicate that air harbors a unique community that may originate from a variety of niches and is shaped by selective forces in the air environment. These findings will aid in formulating public polices guiding the quantification and measurement of indoor air composition for environmental and human health.

## Materials and Methods

### 1. Samples and DNA extraction

#### Air samples

We collected indoor air filters installed in two shopping centers at locations 1 and 2 in Singapore (Figure 1A), between March and April of 2005, for isolation of airborne microbes. The two sites are located 6.7 Km apart from one another; location 1 is situated along the Singapore River, which is connected to the Marina Bay ∼960 meters down stream, and location 2 is ∼1000 meters away from the east coast of Singapore. The retail areas in locations 1 and 2 are 23,111 and 52,144 square meters, respectively. The air supply to each building comprised 80% recycled air and 20% fresh air, processed in air handling units (AHU) to remove airborne dust and lower the temperature. In the AHU, the air flow velocity was on average 1.5 meters per second through the air filters (Figure 1B). There are 15 and 40 AHUs in location 1 and 2 respectively. On average, each AHU has 10–15 air filters mounted in parallel. Panel filters, NGB 290 (Libeltex, Belgium) (Figure 1C), were used in these buildings. The dimensions of the filter are 60×50×2 cm^3^, and the arrestance efficiency is 90% for 1 µm particles. The panel filters were installed for air circulation operation 14 hours per day, and for a total of 90 days before collection. Approximately 2 million cubic meters of air passed through each of the panel filters during the sampling period.

Three pieces of air filters from each site (6 million cubic meters of air per location) were soaked and washed with 10 liters of PBS buffer. The PBS contained no detectable bacterial contamination as determined by PCR using bacterial 16S specific primers (data not shown). The suspension was then filtered through Whatman® filter paper (#114) to remove big particles. We also tested Whatman 3MM Chr filter paper (Cat#3030917) for removing the dusts. Although the flow through was much clearer, the microbial yield was quite low. In both cases, the filtrates were then concentrated by 0.2 µm tangential flow filters (Amersham, CFP-2-E-4A). The microbes were pelleted by centrifugation (9,500 *g*) at 4°C for 30 min. The pellets were resuspended in 10 ml of PBS and stored at −80°C.

Microbial genomic DNA was extracted using the PUREGENE® DNA PURIFICATION KIT (Cat No. D-6000A, Gentra Systems, USA), following the manufacturer's protocol. Briefly, 500 µl of microbial pellet suspension were pelleted in a bench top centrifuge at full speed for 5 seconds. The pellets were resuspended in 600 µl of Cell Lysis Solution and incubated at 80°C for 5 min to lyse cells. The sample was further digested with 3 µl of RNAse A Solution at 37°C for 1 hour. Proteins were precipitated by adding 200 µl of Protein Precipitation Solution and spinning at full speed for 1 min at 4°C. The supernatants were transferred into a clean tube and mixed with 600 µl isopropanol. DNA was pelleted by spinning at full speed (13,200 rpm) for 1 min at 4°C. The DNA pellets were washed with 500 µl 70% ethanol and dissolved in 50 µl of DNA Hydrate Solution by incubating at 37°C for 1 hour. Typically, the yield of genomic DNA was 1 µg from one preparation.

The air sample genomic DNA prepared using a DNA Purification Kit (PUREGENE) still contained black particles from the air filters, which were found to be inhibitory to PCR and restriction enzyme digestion. We further used electrophoresis to separate the insoluble particles from the genomic DNA in a 1% agarose gel. The genomic DNA was excised from the gel and purified using the Qiaquick Gel Extraction Kit (Qiagen), following the protocol of the manufacturer.

#### Soil samples

Soil samples were collected from the surface of open fields near locations 1 and 2, respectively. Plants and large sand particles were removed by passing through mesh sieves. The size selected soil samples were extracted directly using the PUREGENE® DNA Purification Kit (Cat No. D-6000A, Gentra, USA). The humic acids contamination was removed by 0.7% gel electrophoresis. The genomic DNA was excised from the agarose gel and purified by Qiagen gel extraction kit following the manufacturer's protocol. Typically, 300 mg of soil could yield 300–600 ng of genomic DNA.

#### Water samples

The Singapore River is connected to Marina Bay at the southeast coast of Singapore. Essentially, it is an extension of the bay and contains salt water. Twenty liters of water were collected from the Singapore River right next to the shopping mall at location 1. The water was filtered through Whatman paper (#113), and further concentrated by tangential filtration using 0.2 µm filter column (Amersham, CFP-2-E-4A). The concentrated microbial fraction was centrifuged (9,500 g) for 30 min at 4°C. The microbial pellet was extracted for DNA using the PUREGENE® DNA Purification Kit (Cat No. D-6000A, Gentra, USA). The DNA yield is roughly 3 µg from 20 liters of water.

#### Floor dust samples

In the location-2 building, the open floors are daily cleaned with high hygiene standard. Four (4) dead corners (corners next to escalators and open to all floors) in the building were identified with obvious accumulation of dust, representing average sedimentation of air suspension over times. Sterile Kimsweeper tissues were used to sweep the floor to collect the dust, and kept in sterile plastic bags. Dust samples of 3 dead corners immediately outside of the building were also collected similarly. In the lab, dust containing tissues were dissolved in 10 ml of PBS buffer followed by centrifugation to pellet the microbes. The microbial pellet was extracted for DNA using the PUREGENE® DNA Purification Kit (Cat No. D-6000A, Gentra, USA).

#### Human fluid samples

Human nasal swab was collected and re-suspended in 50ul PBS buffer. 2 µl of the solution was directly used for broad range 16S rDNA PCR analysis. Approximately 50 µl of human saliva was collected. 2 µl of the homogenized solution was directly used for broad range 16S rDNA PCR analysis.

### 2. Growth rate assessment of air filter-trapped microorganisms

Filter samples collected from the two locations were tested for growth rate. Sections of the filters (2 cm×2 cm) were soaked in 10 ml PBS and shaken for 15 min at room temperature. The filters were placed in 50 ml syringes, and the liquid forced out using the plunger. From this sample 10 µl was taken for direct counts using an epifluorescent microscope. First, 1 ml of 2% paraformaldehyde was added to the 10 µl samples, which were then incubated at room temperature for 1 hour. The volume was increased to 5 ml in sterile water and the samples were deposited on 0.2 µm filters by vacuum filtration. The filters were stained with 100 µl 5× SYBR Gold (Molecular Probes) for 10 min in the dark, then rinsed in 100 µl H_2_O and mounted on glass slides. Bacterial counts were performed with Image-Pro Plus software (http://www.jknelectronics.com/ippage.htm).

From the 10 ml filtered microbial suspension, 1 ml for each of the three samples was incubated for 1 hour at room temperature in the presence of either 20 nM ^3^H-Leucine or 20 nM ^3^H-Thymidine (Amersham Biosciences). The incubation was terminated by addition of 75 µl 100% trichloroacetic acid (TCA). Blank reactions were set up by adding the TCA at the beginning of the incubation with the radioactive tracer. All reactions were chilled on ice for 10 min, and then pelleted at full speed in a microcentrifuge at room temperature for 5 min. The supernatants were aspirated, and the pellets resuspended in cold 5% TCA, vortexed, and pelleted again as above. This step was repeated with the substitution of 80% ethanol for TCA, and finally the pellets were resuspended in 1 ml scintillation fluid (Ultima Gold) and counted for 3 min in a scintillation counter. The amount of radiation incorporated into newly synthesized DNA was determined by subtracting the average radiation (dpm) in the controls from the average dpm found in the samples. The counting efficiency of the scintillation counter was first determined and used to calculate pmol thymidine incorporated into DNA ml^−1^ hr^−1^ ([adjustment for counting efficiency; 1 dpm per 0.85 cpm]×[1 Ci per 2.22×10^12^ dpm]×[1 mmol per 84 Ci]). Each sample was counted for 3 min. For each sample the 3 replicate counts were averaged and “Carry-over Controls” were subtracted from this value to account for unincorporated radiation. This value was converted to pmol thymidine incorporation cell^−1^ hr^−1^, using the mean direct count data (Table S1).

Based on the number of cells in the filters and the ^3^H incorporation counts, the growth rates of microbial cells washed off from the filters were determined using standard conversion values. The growth rate of cells in sample 1 had an estimated doubling time of 244 (leucine) to 577 (thymidine) days. The incorporations of both leucine and thymidine in samples 2 and 3 were so low that no estimation of growth rates could be determined (i.e., the dpms in the control were equal to or greater than those observed in the samples). Overall, these results suggest essentially no growth of cells in all of the samples (Table S2).

### 3. Cultivation and identification of air filter-trapped Microorganisms

Airborne microbial concentrates in PBS buffer were diluted in 0.9% saline and plated in series on yeast peptone agar for fungal growth at 28°C, and tryptone soy agar and plate count agar for bacterial growth at 37°C. Visibly different colonies based on morphology were picked and streaked on nutrient agar for taxonomic identification.

All the bacterial isolates were Gram stained and then subjected to standard analyses for classification as Gram positive (Catalase and Coagulase tests) and Gram negative (Oxidase test). After these 3 preliminary biochemical tests, a semi-automated bacterial identification instrument (VITEK Systems Inc., USA) was used with the programs for Vitek Bacillus Identification (BAC), the Gram Positive Identification (GPI) and the Gram Negative Identification (GNI) accordingly. For optimal conditions, Gram-positive isolates were diluted in saline to a turbidity concentration of 0.5 McFarland standard and Gram-negative isolates diluted to 1 McFarland standard or their optical equivalents, prior to loading onto the GPI and GNI test cards. Subsequently, these cards were incubated at 35.4°C +/− 2°C and processed automatically by the VITEK systems. Fungal and streptomycete isolates were identified microscopically after staining with lactophenol cotton blue (Sigma) and Gram reagents, respectively.

307 bacterial isolates were classified based on morphology, Gram-staining and standard microbiological tests. 58% of the isolates were Gram-positive and 42% were Gram-negative. A collection of 74 isolates was further characterized using the VITEK system, of which 60 (81%) could be assigned to known taxonomic units of bacteria at genus level (Table S3). The methods used for isolation did not yield abundant fungal isolates. Only 3 *Penicillium* isolates were obtained.

### 4. Broad range 16S rDNA sequence analyses

#### 16S clone library Construction

The 16S rDNA was amplified from purified genomic DNA using bacterial universal primers; Bact-8F (5′-AGAGTTTGATCCTGGCTCAG-3′) and Bact-1391R (5′-GACGGGCGGTGTGTRCA-3′) [30]. Reaction conditions were as follows: 5.0 µl 10× AccuPrime™ PCR Buffer II (Invitrogen), 1.0 µl forward primer and 1.0 µl reverse primer (10 µM each), 0.2 µl AccuPrime™ *Taq* High Fidelity (5 U/µl; Invitrogen) and 1.0 µl of template genomic DNA (10 ng) in a total volume of 50 µl. The following cycling parameters were used: 5 min of initial denaturation at 95°C followed by 20 cycles of denaturation (30 s at 95°C), annealing (30 s at 56°C), and elongation (90 s at 72°C), with a final extension at 72°C for 8 min. Appropriately sized PCR products were agarose gel purified and cloned into the pCR-Blunt II-TOPO vector (Invitrogen). The libraries were transformed into TOP10 electrocompetent cells (Invitrogen) according to manufacturer's protocol.

#### Sequencing and sequence data processing

16S rDNA clones were bidirectionally sequenced according to standard protocols (www.jgi.doe.gov). Paired reads were assembled using phrap and all clones that failed to assemble, formed contigs less than 1250 bp in length or contained less than 80% Q20 bases (or 70% for nasal, saliva and dust samples) were removed from further processing. These clone sequences were then submitted for alignment by the NAST aligner at greengenes (greengenes.lbl.gov) [31]. During this process, any sequences that failed to align to known 16S sequences were removed. The aligned sequences were then checked for chimeric clones using the Bellerophon 3 server at greengenes [32] and likely chimeras were removed from further analysis. The percentage of chimeric sequences in each library was 0.1% or less for the air libraries and 5–10% for soil and water libraries. A total of 2659, 3063, 2408, 2648, and 2941 high-quality 16s rDNA sequences were generated from 16S rDNA clone libraries constructed from samples collected from air filters at locations 1 and 2, soils from locations 1 and 2, and water from the Singapore River near location 1. A total of 984, 688, 565, and 624 sequences were generated from the nasal swab, saliva, and indoor and outdoor dust samples. Cluster information was entered into the EstimateS program (Version 7, R. K. Colwell, http://purl.oclc.org/estimates) for rarefaction curve generation and species richness was estimated with the Chao1 estimator.

#### Phylotype Determination

Cluster representatives were chosen based on phrap quality scores to use the highest quality sequences for further analysis; for large clusters, multiple representatives were chosen. These sequences were then BLAST against an in-house phylogenetically classified 16S sequence database based on greengenes [33], and clusters with hits >1000 bp and >95% identity were assigned to the corresponding phylogenetic group. All other sequences were designated unknown.

### 5. Metagenomic DNA shotgun sequencing analyses

#### Genomic DNA library construction

Approximately 1 µg of genomic DNA from each air sample was partially digested with *Alu*I (Fermentas, 0.1unit/µg DNA) at room temperature for 10 min. The partially digested genomic DNA fragments were loaded on a 0.7% agarose gel for electrophoresis. The 1–2 kb DNA fragments were excised and purified by QIAquick Gel Extraction Kit. The purified DNA fragments (100–500 ng) were blunted at both ends by Klenow enzyme (Fermentas) and de-phosphorylated by alkaline Phosphatase (New England Biolabs). The DNA inserts were ligated with *Eco*RV-digested pZErO-1 vector (Invitrogen) using T4 DNA ligase (Invitrogen) at 16°C overnight. The ligation products were transformed by electroporation into Electrocomp TOP10 cells (Invitrogen). The bacteria were plated on imMedia Zeo Agar plates (Invitrogen). Alternatively, ligations were done using pCR-Blunt II-TOPO (Invitrogen) cloning vector and transformed into Electrocomp TOP10 cells (Invitrogen) for selection by Kanamycin. Colonies were picked by Q-Bot (Applied Biosystem) for sequencing.

#### DNA sequencing and assembly

Raw sequence reads were generated using ABI3730 DNA analyzers, and subjected to base calling and vector/adaptor trimming using PHRED [34], [35]. We used PHRED score 15 as cutoff value for quality sequences because the most meaningful annotation analysis is BLASTX that has high tolerance to minor sequence errors. The average length of quality sequence (PHRED score ≥15) reads for further analysis is 532 bp. The statistics of the sequencing data is presented in Table S5. For each independent sample, all reads (from all libraries) were assembled using Phrap (-minmatch 30 -maxmatch 55 -minscore 55) [36]. Contig length and read depth information was extracted from the phrap output to generate Figure S1.

Large contig sequences were individually analyzed using BLASTN to search for best matches in NCBI GenBank nr database. Though high score matches were found, the alignments of the contig sequences to subject sequences in the database were often patchy, suggesting that they are closely related, but were not derived from the same genome. Two examples are shown in Figure S2.

#### Validation of air contig sequences

Large contig sequences from the two air libraries were selected for validation by semi-quantitative PCR. Contig specific PCR primers were designed to generate amplicon products around 200 bp for detection. Universal 16S rDNA primers were used as quantitative control. The quantity of input DNA templates for PCR from each environmental sample was adjusted based on the relative 16S rDNA PCR result. The PCR reaction conditions were as follows: 2.5 µl 10× Taq Buffer with KCl (Fermentas), 1.5 µl MgCl2 (25 mM, Fermentas), 0.5 µl dNTP mix (10 mM, eppendorf), 1.25 µl forward primer and 1.25 µl reverse primer (10 µM each), 0.25 µl Taq DNA polymerase (5 U/µl; Fermentas) and 1–10 ng of template genomic DNA (normalized by comparing the bands of 16S PCR products of different environmental samples) in a total volume of 25 µl. The following cycling parameters were used: 3 min of initial denaturation at 94°C followed by 35 cycles of denaturation (30 s at 94°C), annealing (30 s at 55°C), and elongation (30 s at 72°C), with a final extension at 72°C for 6 min. The PCR results are shown in Figure S3.

### 6. DNA sequence homology analyses

#### BLASTN to rDNA database

To identify 16S rRNA genes within the genomic shotgun sequence data, vector sequence and low quality sequence with phred scores less than 15 was trimmed from the ends of all reads. The resulting sequences were then subjected to a BLASTN search against an in-house ribosomal RNA database based on Greengenes (greengenes.lbl.gov) [33] with an e-value threshold of 10^−10^. Hits to non-rRNA features such as internal tRNAs were removed manually. The taxonomic groups identified by this analysis are presented in Table S6.

#### BLASTX to GenBank nr database

Quality-trimmed reads as described above were subjected to a BLASTX search against the GenBank nr database with an e-value threshold of 10^−8^. Gene IDs for all hits were collected and phylogenetically placed with an in-house script, TaxBreak. The taxonomic units assigned for each of the sequences are summarized in Table S6.

#### BLASTN to GenBank nt database

Contigs from air assemblies were subjected to a BLASTN search against the GenBank nt database with an e-value threshold of 10^−10^. Hits ≥100 bp and ≥80% identity were analyzed for potential pathogens and commensal organisms.

### 7. Metagenomic gene function annotation

#### Protein prediction

All assembled contigs as well as all singlet reads that failed to assemble were annotated using Fgenesb (www.softberry.com). Proteins were predicted using general open reading frame (ORF) prediction parameters. In total 34,984 and 44,021 ORFs were predicted for the Air-1 and Air-2 libraries, respectively.

#### Environmental Gene Tag (EGT) analysis

The predicted ORF sequences were subjected to BLASTP against the STRING database (http://string.embl.de/) with an e-value of 10^−8^ and assigned to the COG or NOG of the best hit. Proteins that did not have hits were then subjected to BLASTP against the NCBI GenBank nr database and annotated with the best hit. The abundance levels of COGs and NOGs within the air data were compared with previously published environmental datasets using Environmental Gene Tag (EGT) analysis [37]. While the assembled data allows for more accurate gene predictions, the parameter of interest is the number of independent clones encoding homologs of a given functional group. To approximate this, each ORF assigned to a COG was weighted by the number of clones contributing to the contig and divided by the length of the contig to account for contig depth. The total number of clone-weighted homologs was then added up for each COG, and, to normalize for sample size, divided by the total number of clone-weighted homologs for all COGs. This gave the fraction of proteins with COG assignments that were assigned to that particular COG. Finally, when comparing among samples, this number was normalized by the sum across all samples, so that overrepresentation could be compared among different COGs.

#### Supplementary KEGG analysis

The Fgenesb-predicted ORF sequences from the two air samples in this study, and other environmental sequences from other data sources [37], [38] such as seawater, soil and whale fall were subjected to BLASTP against the KEGG database (http://www.genome.ad.jp/kegg/). At a cutoff of 60 bits, each environmental genomic sequence was mapped to at most one protein in the KEGG database (Table S8 in SI).

The frequency of a KEGG pathway hit by an environmental sequence cluster is calculated by the number of individual sequences divided by the total number of sequences in each environmental dataset. The representations of KEGG pathways by the 2 air and 3 other environmental datasets were compared using a two-dimensional matrix clustering (Table S9 in SI). Hierarchical clustering was performed using complete linkage clustering with uncentered correlation as the similarity metric in the CLUSTER package [39].

## Supporting Information

Figure S1 Length vs. depth plot of all the contigs
(0.08 MB DOC)

Figure S2 BLAST analysis of large air sequence contigs
(0.09 MB DOC)

Figure S3 Validation of relative abundance of air sequence contigs in different environments by semi-quantitative PCR
(0.26 MB DOC)

Table S1 Direction of counts and 3H feeding of air filter associated microbes
(0.03 MB DOC)

Table S2 Cell doubling time (days) estimated by two isotope feeding assays
(0.03 MB DOC)

Table S3 Bacteria isolates from filters identified by culture-based assay
(0.04 MB DOC)

Table S4 Bacterial phylotypes identified by 16S rDNA clone sequence analysis
(0.18 MB DOC)

Table S5 Library sequences and assembly statistics
(0.12 MB DOC)

Table S6 16S phylotypes identified by BLASTN analysis and taxonomic units assigned by BLASTX analysis of shotgun genomic DNA sequences
(0.08 MB DOC)

Table S7 Pathogen-related sequences
(0.11 MB DOC)

Table S8 Mapping environmental ORFs to KEGG database
(0.03 MB DOC)

Table S9 KEGG pathways overrepresented in air
(0.04 MB DOC)
