# Supplementary material for: The Airborne Metagenome in an Indoor Urban Environment
Source: PLoS One. 2008 Apr 2;3(4):e1862. doi: 10.1371/journal.pone.0001862 (PMC2270337; doi:10.1371/journal.pone.0001862)
Supplement: Figure S1 — Length vs. depth plot of all the contigs (0.08 MB DOC) [file pone.0001862.s001.doc]

**Supplement Figures**


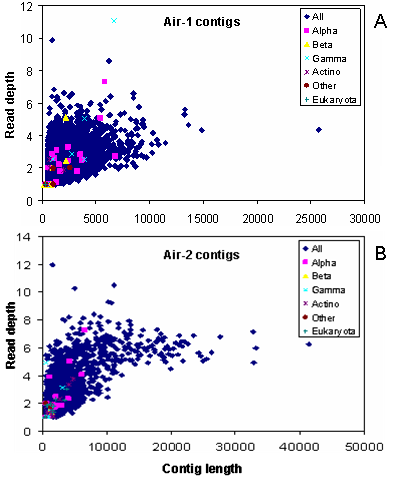


**Figure S1.** Length vs. depth plot of all the contigs in the **A,** Air-1 and **B,** Air-2 assemblies. Contigs containing 16S rRNA genes are colored according to phylogenetic group.
