# Supplementary material for: The Airborne Metagenome in an Indoor Urban Environment
Source: PLoS One. 2008 Apr 2;3(4):e1862. doi: 10.1371/journal.pone.0001862 (PMC2270337; doi:10.1371/journal.pone.0001862)
Supplement: Figure S2 — BLAST analysis of large air sequence contigs (0.09 MB DOC) [file pone.0001862.s002.doc]

**Supplement Figures**

**
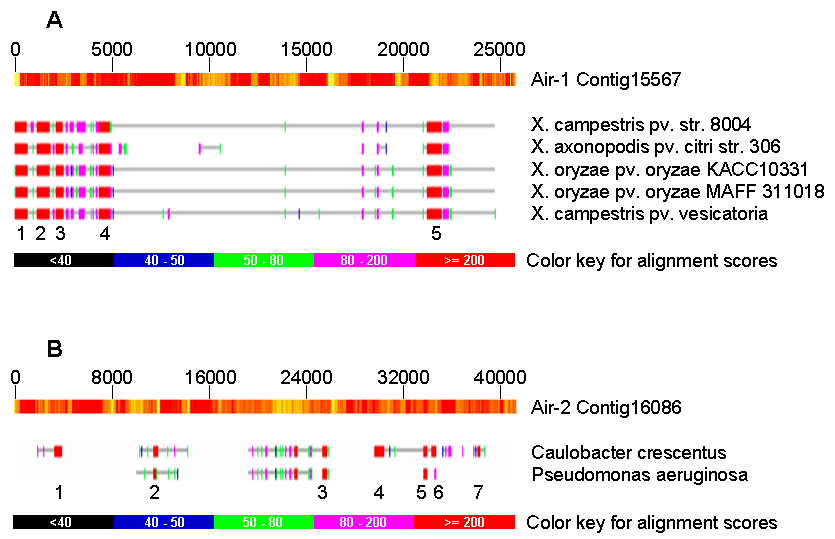
**

**Figure S2.** BLAST analysis of large air sequence contigs

Large sequence contigs were selected for BLASTN in GenBank nr database. The heat maps (top bar) of the contigs reflect the contig quality. The red color indicates high quality regions, while the yellow color for lower quality regions. **A.** Contig Air-1_15567 has 25729 bp. It was matched in patches to various genomic DNA sequences of *Xanthomonas* species. The 5 high score alignment regions (in red color) are in 82-90% identity to subject sequences, which represent the following protein coding sequences: 1. 50S ribosomal protein L20; 2. phenylalanyl-tRNA synthetase alpha chain; 3. phenylalanyl-tRNA synthetase beta chain; 4. integration host factor alpha chain; 5. conserved hypothetical protein. **B.** Contig Air-2_16086 is 41420 bp in length. It best matched in patches to a number of *Caulobacter* and *Pseudomonas* genome sequences. The 7 high score alignment regions represent coding sequences for 1.DNA-binding response regulator; 2. AcrB/AcrD/AcrF family protein; 3. methyl-accepting chemotaxis protein McpA; 4. photosynthesis protein modulator; 5. ferredoxin A; 6. conserved hypothetical protein; and 7. peptidase M1 family protein. Though the BLAST scores and e-values for these matches are high, the structure of the alignments clearly suggested that the contig sequences represent very different genome sequences than the matched ones.
