# Supplementary material for: The Airborne Metagenome in an Indoor Urban Environment
Source: PLoS One. 2008 Apr 2;3(4):e1862. doi: 10.1371/journal.pone.0001862 (PMC2270337; doi:10.1371/journal.pone.0001862)
Supplement: Figure S3 — Validation of relative abundance of air sequence contigs in different environments by semi-quantitative PCR (0.26 MB DOC) [file pone.0001862.s003.doc]

**Supplement Figures**

**
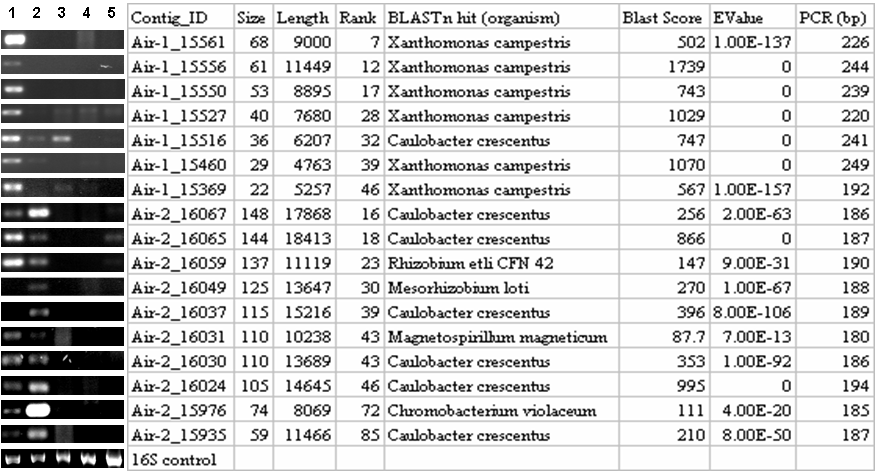
**

**Figure S3.** Validation of relative abundance of air sequence contigs in different environments by semi-quantitative PCR

DNA templates of PCR were from the following environmental samples: **1.** Air-1; **2.** Air-2; **3.** Soil-1; **4.** Soil-2; **5.** Water (Singapore River water sample). The input DNA templates for PCR were adjusted accordingly based on 16S rDNA control. These PCR results clearly suggested that the contig sequences abundant in the air samples were poorly represented in nearby terrestrial and aquatic environmental samples.
