# Supplementary material for: The Airborne Metagenome in an Indoor Urban Environment
Source: PLoS One. 2008 Apr 2;3(4):e1862. doi: 10.1371/journal.pone.0001862 (PMC2270337; doi:10.1371/journal.pone.0001862)
Supplement: Table S1 — Direction of counts and 3H feeding of air filter associated microbes (0.03 MB DOC) [file pone.0001862.s004.doc]

**Supplement Table**

**Table S1.** Direction of counts and ^3^H feeding of air filter associated microbes

| **Sample** | **Cell counts** | **^3^H-Leucine dpm** | **^3^H-Thymidine dpm** |
| --- | --- | --- | --- |
| Air-1 | 2.84 x 10^8^ cells ml^-1^ | 1.59 x 10^5^ | 3.31 x 10^3^ |
| Air-1 blank | * | 5.20 x 10^3^ | 1.57 x 10^3^ |
| Air-2 | 2.90 x 10^8^ cells ml^-1^ | 1.45 x 10^3^ | 6.05 x 10^2^ |
| Air-2 blank | * | 1.81 x 10^3^ | 5.05 x 10^2^ |

* Blanks were treated with trichloroacetic acid to kill cells before incubation with radioactive tracer.
