# Supplementary material for: The Airborne Metagenome in an Indoor Urban Environment
Source: PLoS One. 2008 Apr 2;3(4):e1862. doi: 10.1371/journal.pone.0001862 (PMC2270337; doi:10.1371/journal.pone.0001862)
Supplement: Table S2 — Cell doubling time (days) estimated by two isotope feeding assays (0.03 MB DOC) [file pone.0001862.s005.doc]

**Supplement Table**

**Table S2.** Cell doubling time (days) estimated by two isotope feeding assays

| **Sample** | **Leucine feeding assay** | **Thymidine feeding assay** |
| --- | --- | --- |
| Air-1 | 244 | 577 |
| Air-2 | No detectable growth | No detectable growth |
