# Supplementary material for: The Airborne Metagenome in an Indoor Urban Environment
Source: PLoS One. 2008 Apr 2;3(4):e1862. doi: 10.1371/journal.pone.0001862 (PMC2270337; doi:10.1371/journal.pone.0001862)
Supplement: Table S3 — Bacteria isolates from filters identified by culture-based assay (0.04 MB DOC) [file pone.0001862.s006.doc]

**Supplement Table**

**Table S3.** Bacteria isolates from filters identified by culture-based assay

| **Cultured microbial isolates*** | **Air-1** | **Air-2** |
| --- | --- | --- |
| Acinetobacter | 1 | 1 |
| Bacillus | 4 | 6 |
| Corynebacterium | 2 | 2 |
| Brevundimonas | 2 | 2 |
| Nonfermenting Gram Negative spp | 1 | 1 |
| Staphylococcus | 5 | 7 |
| Stenotrophomonas | 5 | 2 |
| Streptococcus | 1 | 1 |
| Streptomyces | 17 | 0 |
| Total (10 genera) | 38 | 22 |

* Colonies were chosen for analysis based on morphology and thus total counts are not representative of abundance.
