# Supplementary material for: The Airborne Metagenome in an Indoor Urban Environment
Source: PLoS One. 2008 Apr 2;3(4):e1862. doi: 10.1371/journal.pone.0001862 (PMC2270337; doi:10.1371/journal.pone.0001862)

**Supplement Table**

**Table S4.** Bacterial phylotypes identified by 16S rDNA clone sequence analysis (>97%) in air and nearby soil and aquatic environments. Genera listed in boldface are those that appeared among the cultured isolates.


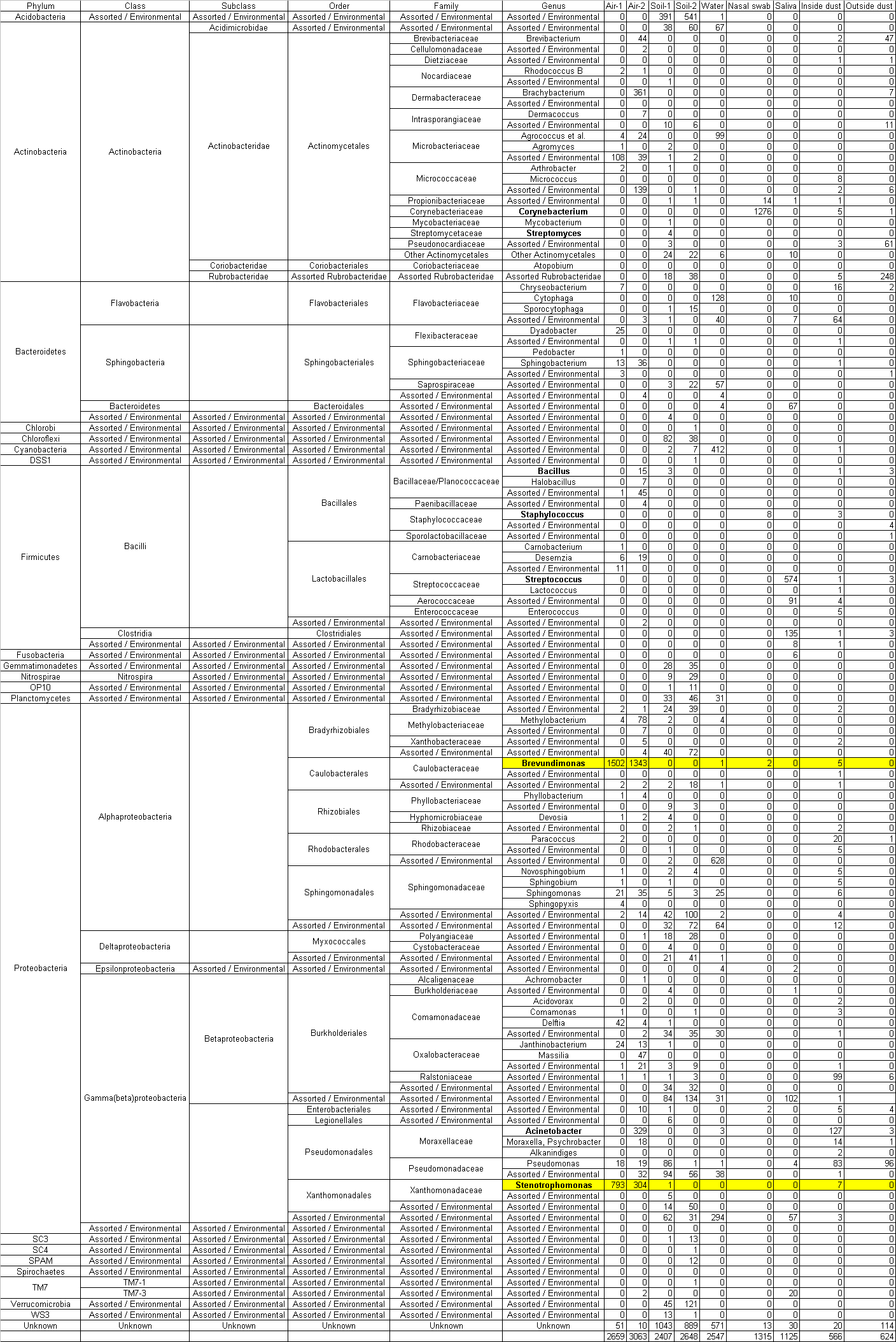

Supplement: Table S4 — Bacterial phylotypes identified by 16S rDNA clone sequence analysis (0.18 MB DOC) [file pone.0001862.s007.doc]
