# Supplementary material for: The Airborne Metagenome in an Indoor Urban Environment
Source: PLoS One. 2008 Apr 2;3(4):e1862. doi: 10.1371/journal.pone.0001862 (PMC2270337; doi:10.1371/journal.pone.0001862)
Supplement: Table S5 — Library sequences and assembly statistics (0.12 MB DOC) [file pone.0001862.s008.doc]

**Supplement Table**

**Table S5:** Library sequences and assembly statistics
