# Supplementary material for: The Airborne Metagenome in an Indoor Urban Environment
Source: PLoS One. 2008 Apr 2;3(4):e1862. doi: 10.1371/journal.pone.0001862 (PMC2270337; doi:10.1371/journal.pone.0001862)

**Supplement Table**

**Table S6:** 16S phylotypes identified by BLASTN analysis and taxonomic units assigned by BLASTX analysis of shotgun genomic DNA sequences


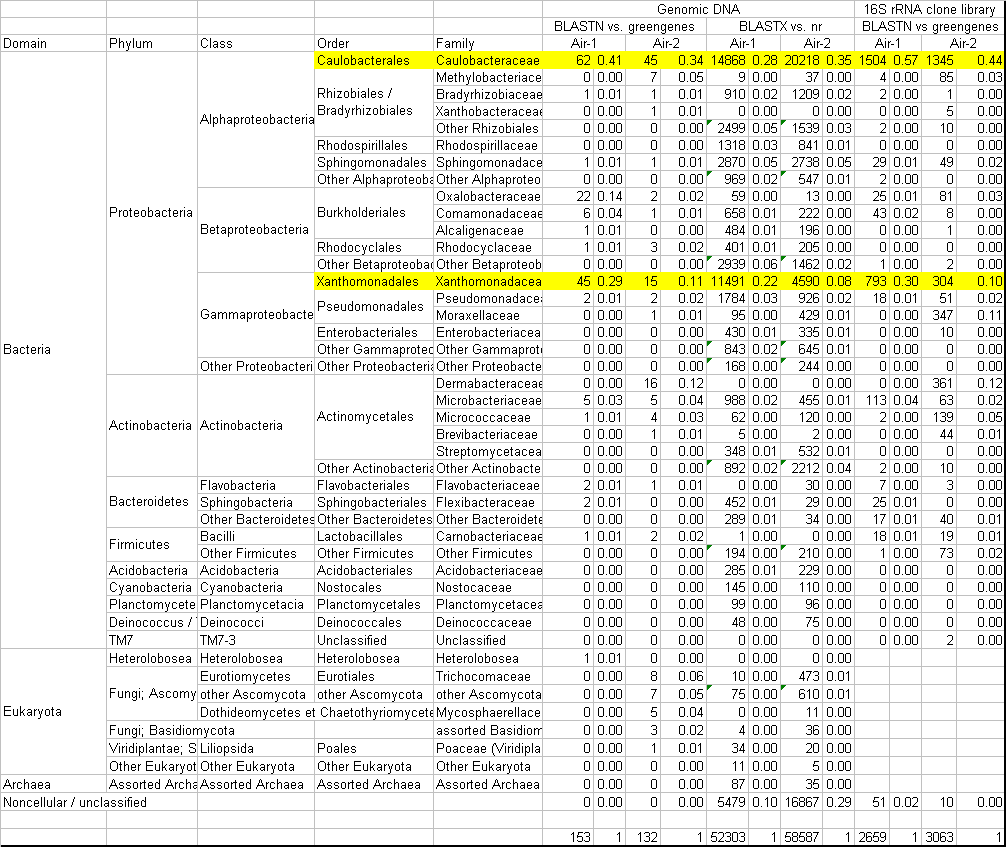

Supplement: Table S6 — 16S phylotypes identified by BLASTN analysis and taxonomic units assigned by BLASTX analysis of shotgun genomic DNA sequences (0.08 MB DOC) [file pone.0001862.s009.doc]
