# Supplementary material for: The Airborne Metagenome in an Indoor Urban Environment
Source: PLoS One. 2008 Apr 2;3(4):e1862. doi: 10.1371/journal.pone.0001862 (PMC2270337; doi:10.1371/journal.pone.0001862)
Supplement: Table S7 — Pathogen-related sequences (0.11 MB DOC) [file pone.0001862.s010.doc]

**Supplement Table**

**Table S7:** Pathogen-related sequences

16S rDNA clone sequences and shotgun genomic DNA sequences were matched to known virulent pathogens, opportunistic pathogens, and human commensal bacteria by BLASTN (e-val ≤ 10^-8^). The numbers are the number of hits to each bacterial species.

| **Species** | **16S rDNA** | | **Genomic DNA** | |
| --- | --- | --- | --- | --- |
|  | **Air-1** | **Air-2** | **Air-1** | **Air-2** |
| **Virulent pathogens** | | | | |
| Bordetella bronchiseptica strain RB50 | 0 | 0 | 51 | 23 |
| Bordetella parapertussis strain 12822 | 0 | 0 | 17 | 2 |
| Bordetella pertussis strain Tohama I | 0 | 0 | 12 | 7 |
| Brucella abortus biovar 1 str. 9-941 | 0 | 0 | 1 | 1 |
| Brucella melitensis 16M | 0 | 0 | 0 | 1 |
| Brucella suis 1330 | 0 | 0 | 6 | 4 |
| Burkholderia mallei ATCC 23344 | 0 | 0 | 11 | 6 |
| Burkholderia sp. 383 | 0 | 0 | 45 | 0 |
| Clostridium tetani E88 | 0 | 0 | 1 | 0 |
| Corynebacterium diphtheriae gravis NCTC13129 | 0 | 0 | 0 | 3 |
| Cryptosporidium parvum | 0 | 0 | 2 | 3 |
| Escherichia coli CFT073 | 0 | 0 | 0 | 2 |
| Escherichia coli O157:H7 | 0 | 0 | 1 | 2 |
| Escherichia coli UTI89 | 0 | 0 | 0 | 1 |
| Leishmania major strain Friedlin | 0 | 0 | 0 | 1 |
| Mycobacterium tuberculosis CDC1551 | 0 | 0 | 2 | 9 |
| Salmonella enterica subsp. enterica serovar Choleraesuis str. SC-B67 | 0 | 0 | 0 | 1 |
| Salmonella typhimurium LT2 | 0 | 0 | 0 | 1 |
| Shigella flexneri 2a str. 2457T | 0 | 0 | 0 | 10 |
| **Opportunistic pathogens** | | | | |
| Acinetobacter baumannii str. AYE | 0 | 0 | 0 | 1 |
| Acinetobacter schindleri | 0 | 32 | 0 | 0 |
| Acinetobacter sp. ADP1 | 0 | 0 | 1 | 45 |
| Aspergillus fumigatus Af293 | 0 | 0 | 1 | 11 |
| Bacillus cereus | 0 | 2 | 0 | 1 |
| Bacteroides fragilis NCTC 9343 | 0 | 0 | 0 | 1 |
| Brevundimonas diminuta | 40 | 234 | 26 | 0 |
| Brevundimonas vesicularis | 110 | 481 | 1 | 0 |
| Burkholderia cenocepacia AU 1054 | 0 | 0 | 45 | 0 |
| Burkholderia cepacia | 0 | 0 | 1 | 2 |
| Burkholderia pseudomallei 1710b | 0 | 0 | 6 | 9 |
| Caryophanon sp. oral clone AW086 | 0 | 0 | 0 | 0 |
| Chromobacterium violaceum ATCC 12472 | 0 | 0 | 34 | 30 |
| Corynebacterium jeikeium K411 | 0 | 0 | 2 | 12 |
| Enterobacter aerogenes | 0 | 0 | 0 | 1 |
| Enterobacter hormaechei | 0 | 9 | 0 | 0 |
| Enterococcus faecalis V583 | 0 | 0 | 6 | 0 |
| Haloanella gallinarum | 0 | 3 | 0 | 0 |
| Klebsiella pneumoniae | 0 | 0 | 0 | 3 |
| Massilia sp. VA23069_03 | 0 | 0 | 0 | 0 |
| Massilia timonae | 0 | 47 | 0 | 0 |
| Mycobacterium avium | 0 | 0 | 1 | 10 |
| Mycobacterium avium subsp. paratuberculosis str. K10 | 0 | 0 | 10 | 35 |
| Mycobacterium smegmatis | 0 | 0 | 0 | 9 |
| Nocardia farcinica IFM 10152 | 0 | 0 | 28 | 89 |
| Pseudomonas aeruginosa PAO1 | 0 | 0 | 38 | 54 |
| Ralstonia mannitolilytica | 1 | 0 | 0 | 0 |
| Rickettsia felis URRWXCal2 | 0 | 0 | 0 | 1 |
| Sphingomonas sp. oral clone AV069 | 0 | 0 | 0 | 0 |
| Stenotrophomonas maltophilia | 793 | 304 | 38 | 22 |
| Streptococcus pyogenes MGAS10750 | 0 | 0 | 1 | 0 |
| **Commensals** | | | | |
| Brevibacterium casei | 0 | 3 | 0 | 0 |
| Brevibacterium sp. H15 | 0 | 9 | 0 | 0 |
| Methylobacterium podarium | 0 | 2 | 0 | 0 |
| Enterobacter cloacae | 0 | 0 | 0 | 0 |
| Acinetobacter calcoaceticus | 0 | 0 | 0 | 0 |
| Micrococcus luteus | 0 | 0 | 0 | 21 |
| Propionibacterium acnes KPA171202 | 0 | 0 | 0 | 7 |
| Escherichia coli K-12 MG1655 | 0 | 0 | 0 | 4 |
| Escherichia coli strain LW1655F+ | 0 | 0 | 0 | 1 |
| Bifidobacterium longum NCC2705 | 0 | 0 | 1 | 1 |
| Brevibacterium linens | 0 | 0 | 2 | 1 |
| Bacteroides thetaiotaomicron VPI-5482 | 0 | 0 | 1 | 0 |
