# Supplementary material for: The Airborne Metagenome in an Indoor Urban Environment
Source: PLoS One. 2008 Apr 2;3(4):e1862. doi: 10.1371/journal.pone.0001862 (PMC2270337; doi:10.1371/journal.pone.0001862)
Supplement: Table S8 — Mapping environmental ORFs to KEGG database (0.03 MB DOC) [file pone.0001862.s011.doc]

**Supplement Table**

**Table S8.** Mapping environmental ORFs to KEGG database

|  | **Air-1** | **Air-2** | **Soil** | **Whale**  **Fall** | **Sargasso Sea** |
| --- | --- | --- | --- | --- | --- |
| Predicted ORFs | 34984 | 44021 | 184375 | 122151 | 1001987 |
| ORFs to KEGG  (% of total) | 12370 (35.3%) | 12279 (27.9%) | 49381 (26.8%) | 40537 (33.1%) | 509045 (50.8%) |
