# Supplementary material for: The Airborne Metagenome in an Indoor Urban Environment
Source: PLoS One. 2008 Apr 2;3(4):e1862. doi: 10.1371/journal.pone.0001862 (PMC2270337; doi:10.1371/journal.pone.0001862)
Supplement: Table S9 — KEGG pathways overrepresented in air (0.04 MB DOC) [file pone.0001862.s012.doc]

**Supplement Table**

**Table S9.** KEGG pathways overrepresented in air (>1.5 fold)

Numbers are fraction of total ORFs assigned to each pathway.

| **KEGG pathways** | **Air-1** | **Air-2** | **Soil** | **Whale Fall** | **Sargasso Sea** |
| --- | --- | --- | --- | --- | --- |
| Type IV secretion system | 0.002544 | 0.00293 | 0.000174 | 0.000999 | 0.0002046 |
| Toluene and xylene degradation | 0.000743 | 0.000636 | 0.000168 | 0.000319 | 0.0002844 |
| Biosynthesis of siderophore group nonribosomal | 0.000457 | 0.000386 | 0.000217 | 9.82E-05 | 0.0001737 |
| ABC transporters - Organism-specific | 0.004688 | 0.004316 | 0.0016 | 0.002734 | 0.0026717 |
| Diterpenoid biosynthesis | 0.000286 | 0.000341 | 9.76E-05 | 0.000115 | 0.0001088 |
| Flagellar assembly | 0.007203 | 0.00443 | 0.001589 | 0.004437 | 0.0028244 |
| Type III secretion system | 0.00243 | 0.00159 | 0.000694 | 0.001515 | 0.001029 |
